# Compound drought and heatwave extreme weather events: Mortality risk in individuals with chronic respiratory disease

**DOI:** 10.1097/EE9.0000000000000389

**Published:** 2025-05-01

**Authors:** Austin Rau, Arianne K. Baldomero, Jesse E. Bell, Jared Rennie, Chris H. Wendt, Gillian A. M. Tarr, Bruce H. Alexander, Jesse D. Berman

**Affiliations:** a Division of Environmental Health Sciences, University of Minnesota School of Public Health, Minneapolis, Minnesota; b Pulmonary, Allergy, Critical Care, and Sleep Medicine Section, Minneapolis VA Health Care System, Minneapolis, Minnesota; c Division of Pulmonary, Allergy, Critical Care, and Sleep Medicine, University of Minnesota School of Medicine, Minneapolis, Minnesota; d University of Nebraska, Daugherty Water for Food Global Institute, Lincoln, Nebraska; e Department of Environmental, Agricultural and Occupational Health, University of Nebraska Medical Center, Omaha, Nebraska; f University of Nebraska-Lincoln, School of Natural Resources, Lincoln, Nebraska; g National Oceanic and Atmospheric Administration, National Centers for Environmental Information, Asheville, North Carolina

**Keywords:** Heatwave, Drought, Compounding climate hazard, COPD, Mortality

## Abstract

**Background:**

Compound extreme weather events are severe weather conditions that can jointly magnify human health risks beyond any single event alone. Drought and heatwaves are extreme weather conditions associated with adverse health, but their combined impact is poorly understood.

**Methods:**

We designed a case–crossover study to estimate heatwave-associated mortality stratified by drought conditions in 183,725 US Veteran patients (2016–2021) with chronic obstructive pulmonary disease (COPD). A conditional logistic regression with distributed lag models was applied. Droughts were categorized into binary and categorical metrics, and we further explored the timing of heatwaves as a risk factor.

**Results:**

Our results indicate that drought amplifies heatwaves with hotter temperatures and longer durations during drought conditions, and the percentage of mortality attributable to heatwaves during drought was 7.41% (95% confidence interval [CI]: 2.91, 12.28) compared with 2.91% (95% CI: 0.00, 4.76) for heatwaves during nondrought conditions. Heatwaves that occurred during drought conditions in the late warm season had a larger association with mortality compared with late-season heatwaves during nondrought conditions, 7.41% (95% CI: 1.96, 13.04) of mortality events and 0.99% (95% CI: −1.01, 3.85) of mortality events attributable to these exposures, respectively.

**Conclusion:**

Compound drought and heatwave events trend toward increased mortality risk among patients with COPD and present a growing human health threat under climate change. Existing heat warnings and vulnerability maps may include drought conditions to better capture heat-related public health risks.

**What this study adds**

This study explores the mortality risks during a poorly evaluated compound extreme weather condition of simultaneous drought and heatwaves among individuals with chronic respiratory disease. We highlight the synergistic human health risk from heatwaves during drought conditions using individual-level health data, geocoded households, and spatially resolved meteorology data and drought indices. This study offers novel insights into the health concerns surrounding compound extreme climate events, which are increasing in frequency and should be considered by policymakers and agencies when developing climate action plans and mitigation strategies for extreme weather and health.

## Introduction

The societal and human health mortality and morbidity impacts of climate-related hazards such as heatwaves, wildfires, and drought are globally established.^1–6^ Yet, these hazards seldom occur in isolation and frequently present as compound events occurring simultaneously in the same location,^7,8^ or as cascading events that occur sequentially.^9^ The frequency of compounding climate events is expected to increase under climate change,^7,10–13^ however, little work has described the potential added health risks from compound climate events.^14,15^ The failure to consider public health risks from compound climate events may lead to insufficient preparedness and emergency response.

Extreme heat and drought conditions are independently associated with an increased risk of mortality in the general population.^1,16–18^ An average of 700 heat-related deaths occurred annually in the United States from 2004 to 2018,^19^ while an estimated seven deaths per 10 million people are attributed to each additional extreme heat day per month.^18^ Drought, characterized by abnormally dry conditions, is a slow onset, spatially defuse climate hazard that can synergize with and exacerbate other environmental exposures including ozone, dust, and wildfires.^20–22^ Heatwaves are intensified under drought conditions via a complex land–atmosphere feedback loop,^23^ and compound drought and heatwave events in particular are likely to continue or worsen in the near future.^24–26^

Chronic obstructive pulmonary disease (COPD) is a lung disease characterized by obstructed airflow. Additionally, COPD is a disease sensitive to climate events where extreme heat has been shown to increase the risk of hospitalization and death in afflicted individuals.^27–31^ Other work has found associations between drought conditions and broad categories of respiratory disease morbidity and mortality.^2,16,32,33^ However, the extent to which other extreme weather events such as drought conditions can amplify heat-related morbidity and mortality among individuals with obstructive lung diseases is unknown, despite the increasing risk of these events occurring simultaneously under changing climate.^13,25^ We measured the risk of all-cause mortality due to compound drought and heatwaves in patients diagnosed with COPD using data from the Veterans Health Administration (VHA), 2016–2021. Differences in mortality risk with respect to the timing of heatwaves and droughts were also explored.

## Methods

### Study cohort

We used electronic health records for veterans diagnosed with COPD who died between 2016 and 2021 during the warm season (April–September) in the contiguous United States (N = 183,725). These patients were between the ages of 35 and 100 at initial COPD diagnosis and had two or more clinical encounters (in-patient or outpatient) with an International Classification of Diseases 10th revision codes (ICD-10) for COPD (J40, J41.X, J42, J43.X, and J44.X).^34^ Data including age, self-reported gender and race, and geocoded residential addresses were obtained from electronic records stored in the VHA Veteran Enrollee files, Corporate Data Warehouse.^35^ The study cohort is described in more depth in our previous studies.^31,36^

### Weather data

We assigned daily weather (mean temperature, total precipitation, mean wind speed, and mean specific humidity) to geocoded patient addresses by spatially overlaying home locations with GridMet, a highly resolved weather dataset with a gridded resolution of 4 × 4 km.^37^ Heatwaves were defined as ≥2 consecutive days with a mean daily temperature exceeding the 90th percentile of mean warm season daily temperature based on a 30-year reference distribution (1992–2021) at each patient’s home location.

### Drought data

Droughts were defined using two separate data sources: the United States Drought Monitor (USDM) and the Standardized Precipitation-Evapotranspiration Index (SPEI).^38,39^ The USDM blends physical climate data, drought impacts, and on-the-ground expert observations to classify drought conditions for North America on a weekly basis.^39^ SPEI, a dataset with global spatial coverage, is an extension of the Standardized Precipitation Index that uses precipitation and potential evapotranspiration to classify drought through the impact of increased temperature on water demand.^40^

We acquired weekly USDM data at the county level from the USDM program and the 12-month SPEI at a spatial resolution of 5 km with a monthly temporal resolution from the National Center for Environmental Information.^40^ USDM values included no drought, abnormally dry (D0) and four levels of drought: moderate drought (D1), severe drought (D2), extreme drought (D3), and exceptional drought (D4).^39^ We classified droughts on a month-to-month basis using both a binary (no drought, drought) and a categorical (no drought, moderate drought, severe drought) definition.

To convert weekly USDM data into a monthly level drought indicator, we started by expanding the weekly data into a daily time series assuming each day had the same USDM value during a given week. Next, we assigned each county a daily USDM value (no drought, D0, D1, D2, D3, or D4) using the USDM category with the largest affected land area for each county (the highest category was chosen in the presence of ties). These daily, county-level USDM data were then collapsed into simplified binary and categorical definitions of drought. The binary definition included the USDM levels of no drought (no drought and D0) and drought (D1–D4). The categorical definition included the USDM levels of no drought, moderate drought (D1–D2), and severe drought (D3–D4). Finally, the daily data were aggregated to the monthly level where each month was assigned the most frequent daily USDM drought category (for binary and categorical definitions, respectively) in that month to reflect the long-lasting, chronic nature of drought conditions. For SPEI, we applied a zonal mean to calculate the mean SPEI values in each county per month. The raw SPEI values were converted to USDM equivalent values (e.g., no drought to D4) for ease of comparison,^41^ and then classified into binary and categorical drought definitions described above.

### Study design

A time-stratified case–crossover study design was implemented to determine if heatwave-associated mortality risk was altered by drought conditions in our national COPD patient cohort. In a case–crossover approach, each patient’s date of death is matched with referent days of the same day of week, month, and year as the date of death allowing us to quantify if mortality risk is greater during extreme weather conditions compared with normal conditions. A strength of this self-matched study design is that each person is compared with themselves, thereby controlling for time-invariant individual risk factors (e.g., comorbidities, tobacco use, and socioeconomic status), seasonal, and day-of-week confounding by design.^42–44^

### Statistical analysis

Distributed lag models (DLM) with conditional logistic regression were developed to estimate incidence rate ratios (IRRs) of associations between heatwaves and mortality during both drought and nondrought conditions.^43^ Our primary analysis evaluated the associations between heatwaves and all-cause mortality stratified by drought status using our USDM definition. Data were stratified for (1) binary drought, (2) categorical drought, and (3) early (April–June) and late (July–September) season heatwaves. Stratification by heatwave timing was completed for the binary drought definition. As a secondary analysis, we repeated the stratified data analyses and timing evaluation using the SPEI drought definition as a mechanism to demonstrate the robustness of our results when using a different drought data source and to provide a plausible exposure metric for global drought and heatwave assessments. All models were adjusted for daily time-varying confounders of precipitation and wind speed as linear terms, specific humidity as a natural cubic spline term with five degrees of freedom, and a binary holiday status term for all federally recognized holidays and Easter.

Heatwave associations were estimated at each single lag day from 0 to 1 day before death as well as the cumulative association across the entire lagged period to capture potential delayed health responses from heatwave exposure. The percentage of mortality events attributable to heatwave exposure during the different drought conditions (attributable risk % [AR%]) was calculated for the cumulative lagged periods (Equation 1).

Attributable risk (%) calculation:

$$\begin{array}{l} AR\%=\frac{\left(IRR-1\right)\;}{IRR}\times 100. \end{array}$$ (1)

Two-sided *Z* tests with an *α* level of 0.05 were used to compare differences in stratum-specific associations.^45^ All analyses were completed in R statistical software version 4.1 (R Core Team, Vienna, Austria),^46^ and DLM models were built using the *dlnm* R package.^47^

### Sensitivity analysis

We assessed the sensitivity of our findings to droughts defined by a long-duration criteria where drought events could only be considered if moderate drought or above persisted for at least 5 consecutive months, otherwise, a county would be classified as not experiencing a drought event.^2^ This analysis enabled us to assess only persistent drought events and was completed for both USDM and SPEI binary drought measures. Second, we defined drought conditions using weekly USDM data. This was completed for the binary USDM drought definition where individuals in our study were partitioned into strata of drought based on exposure during their week of mortality. Third, we assessed potential confounding from air pollution by including daily fine particulate matter (PM_2.5_) into our model of binary drought (USDM) for all patients living within a 10-km radius of an Environmental Protection Agency regulatory monitor (n = 20,735).^48^

### Ethics statement

This study was approved by the institutional review boards at the Minneapolis VA Health Care System (11 August 2020; IRB # VAM-20-00583) and the University of Minnesota (2 October 2020; IRB # STUDY00011069).

## Results

### Descriptive statistics

Our study cohort included 183,725 patients with COPD in the VHA system who died during the warm season between 2016 and 2021 (eFigure 1; https://links.lww.com/EE/A344). The mean age was 76.9 years, and patients were predominantly male and of White race (eTable 1; https://links.lww.com/EE/A344). There was a pattern of higher mortality frequencies among patients exposed to both drought and heatwaves with increasing mortality during more severe droughts (Table 1). Among COPD patients exposed to heatwaves during severe drought conditions, 15.3% of patients died compared with 9% of patients who died during heatwaves under nondrought conditions (Table 1). Higher mortality frequencies under simultaneous heatwave and drought conditions were also observed when stratifying the data by timing of heatwaves in the warm season of the year (eTable 2; https://links.lww.com/EE/A344). Similar patterns were observed when using the alternative SPEI drought definitions (Table 1).

**Table 1.** Frequency of deceased COPD patients stratified by heatwave and drought status on the day of death

		Drought classification			
Drought index	Heatwave status	No drought	Any drought	Moderate drought	Severe drought
USDM	Nonheatwaves, n (%)	145,336 (91.0%)	20,768 (86.3%)	16,369 (86.8%)	4,399 (84.7%)
	Heatwaves, n (%)	14,336 (9.0%)	3,285 (13.7%)	2,489 (13.2%)	796 (15.3%)
	Total	159,672	24,053	18,858	5,195
SPEI	Nonheatwaves, n (%)	129,127 (91.5%)	36,977 (87.0%)	25,760 (87.6%)	11,217 (85.6%)
	Heatwaves, n (%)	12,072 (8.5%)	5,549 (13.0%)	3,661 (12.4%)	1,888 (14.4%)
	Total	141,199	42,526	29,421	13,105

Days with both heatwave and drought exposure were hotter than days with only heatwave exposure with an average temperature of 28.3 °C during drought conditions and 27.8 °C during nondrought periods (Table 2). Heatwaves also lasted longer during drought conditions with an average duration of 6.4 days for heatwaves during drought and 4.8 days in the absence of drought (Table 2). The trend of hotter and longer heatwaves during drought conditions was escalated for heatwaves that occurred later in the warm season compared with earlier (eTable 3; https://links.lww.com/EE/A344). Drought defined by SPEI similarly accentuated heatwaves (Table 2). Spatial heterogeneities in both heatwave temperature and duration changes during drought were also observed (eFigure 2; https://links.lww.com/EE/A344).

**Table 2.** Mean heatwave temperature and duration on days of mortality stratified by binary and categorical drought conditions

		Drought classification			
Drought index	Heatwave status/duration	No drought	Any drought	Moderate drought	Severe drought
USDM	Nonheatwave mean temperature, °C (standard deviation)	20.3 (6.4)	21.1 (6.0)	21.0 (6.0)	21.7 (6.1)
	Heatwave mean temperature, °C (standard deviation)	27.8 (2.7)	28.3 (4.2)	28.0 (4.1)	29.1 (4.5)
	Heatwave mean duration days (standard deviation)	4.8 (3.1)	6.4 (5.5)	6.4 (5.9)	6.2 (4.1)
SPEI	Nonheatwave mean temperature, °C (standard deviation)	20.3 (6.3)	21.0 (6.4)	20.7 (6.5)	21.7 (6.2)
	Heatwave mean temperature, °C (standard deviation)	27.5 (2.5)	28.7 (3.8)	28.6 (3.5)	28.9 (4.2)
	Heatwave mean duration days (standard deviation)	4.6 (2.9)	6.1 (4.9)	5.6 (3.9)	7.0 (6.4)

### Heatwave-associated mortality during drought and nondrought conditions

**Figure 1.**
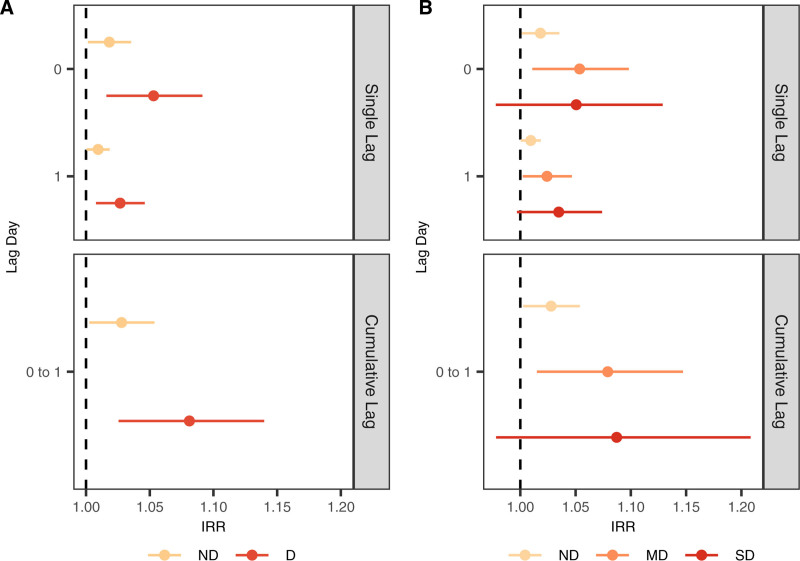
Adjusted incidence rate ratios (IRRs) describing the association between heatwaves and all-cause mortality among patients with COPD stratified by binary (A) and categorical (B) USDM drought definitions. D, drought; MD, moderate drought; ND, no drought; SD, severe drought.

A pattern of increased mortality risk was observed during heatwaves occurring under drought conditions, although the estimates were less precise (i.e., wider confidence intervals [CI]) and stratum-specific differences did not exclude the null at the 95% confidence level. The cumulative heatwave association with mortality during any drought (binary definition) had an IRR of 1.08 (95% CI: 1.03, 1.14) (Figure 1 and eTable 4; https://links.lww.com/EE/A344). Among patients exposed to heatwaves during drought conditions, 7.41% (95% CI: 2.91, 12.28) of deaths were attributable to heatwaves (Table 3). Heatwave-related mortality risks increased as the severity of drought increased from moderate to severe (Figure 1). The cumulative association of heatwaves during severe drought had an IRR of 1.09 (95% CI: 0.98, 1.21) and AR% of 8.26 (95% CI: −2.04, 17.36) compared with IRR of 1.03 (95% CI: 1.00, 1.05) and AR% of 2.91 (95% CI: 0.00, 4.76) when no drought was present (Figure 1, Table 3, and eTable 4; https://links.lww.com/EE/A344).

**Table 3.** Attributable risk (%) for the cumulative (lag 0–1) heatwave associations stratified by USDM drought status (binary and categorical) and by the timing of heatwaves

Strata	AR% (95% CI)
No drought	2.91 (0.00, 4.76)
Any drought	7.41 (2.91, 12.28)
Moderate drought	7.41 (1.96, 13.04)
Severe drought	8.26 (−2.04, 17.36)
Heatwave timing	
Early (no drought)	9.09 (3.85, 13.79)
Early (drought)	8.26 (−3.09, 18.03)
Late (no drought)	0.99 (−1.01, 3.85)
Late (drought)	7.41 (1.96, 13.04)

### Timing differences in heatwave-associated mortality during drought and nondrought conditions

**Figure 2.**
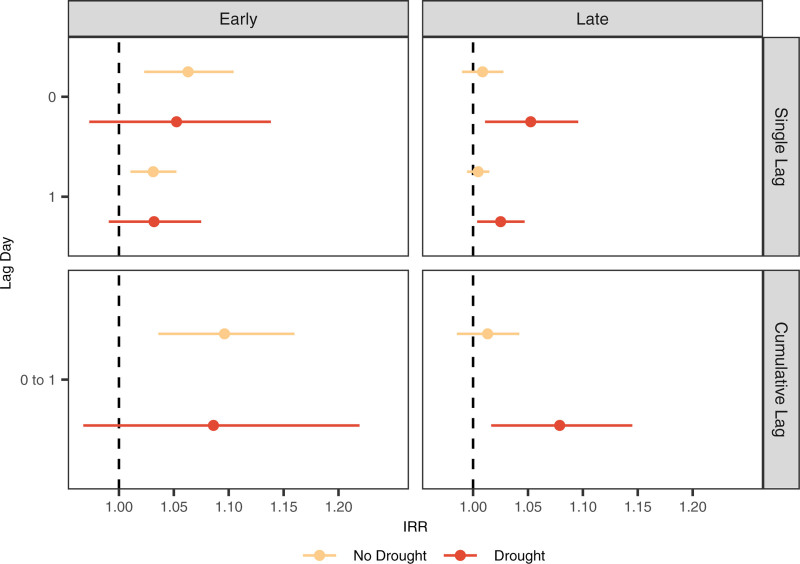
Adjusted incidence rate ratios (IRRs) describing the association between heatwaves and all-cause mortality among patients with COPD stratified by binary USDM drought definitions and timing of heatwaves (early = April–June and late = July–September).

Heatwaves that occurred during drought conditions in the latter half of the warm season had a pattern of larger mortality risks although with less precise estimates, and stratum-specific differences that did not exclude the null at the 95% confidence level (Figure 2, Table 3, and eTable 5; https://links.lww.com/EE/A344). The cumulative mortality risk was IRR: 1.08 (95% CI: 1.02, 1.15) for late-season heatwaves during drought compared with IRR: 1.01 (95% CI: 0.98, 1.04) for late-season heatwaves during nondrought periods (Figure 2 and eTable 5; https://links.lww.com/EE/A344). This difference in risk was also seen in the AR% estimates, AR%: 7.41% (95% CI: 1.96, 13.04) and AR%: 0.99% (95% CI: −1.01, 3.85), respectively, which reveal a substantially larger mortality risk in late-season heatwaves that occur during drought conditions for patients with COPD. Heatwave-associated mortality risks comparing drought and nondrought periods in the early warm season were similar to each other (Table 3 and eTable 5; https://links.lww.com/EE/A344).

### Sensitivity analysis

Using an alternate drought definition with SPEI drought measures revealed minor attenuation of mortality risks compared with drought exposure quantified with USDM data, but our qualitative interpretation remained unchanged. This observation remained true for binary drought, categorical drought, and timing of heatwaves in the year (eTables 6–8 and eFigures 3 and 4; https://links.lww.com/EE/A344). When assessing a longer-term 5-month duration criterion to define drought events, we found heatwave-associated mortality risks during drought were larger compared with our primary analysis (eFigure 5; https://links.lww.com/EE/A344). The cumulative lag IRR for heatwaves during drought events was IRR: 1.12 (95% CI: 1.06, 1.19), a 4% increase in mortality risk compared with our primary analysis. We found no differences between heatwave-associated mortality risk during drought and nondrought periods using SPEI comparing our primary analysis to a 5-month duration criterion for drought (eFigure 5; https://links.lww.com/EE/A344). There were no differences in heatwave-associated mortality risk using weekly versus monthly USDM drought exposure (eFigure 6; https://links.lww.com/EE/A344).

Our models were robust to potential confounding bias by air pollution. In a model adjusted for PM_2.5_ among patients who lived within 10 km of Environmental Protection Agency air monitors, the cumulative lag IRR for heatwaves during drought was IRR: 1.16 (95% CI: 1.03, 1.32) compared with IRR: 1.14 (95% CI: 1.06, 1.23) in a model not adjusted for PM_2.5_ in this same population.

## Discussion

Our evaluation presented a pattern of hotter and longer duration heatwaves occurring in the United States during drought conditions compared with nondrought conditions. Among our population with diagnosed COPD, we estimated an increased heatwave-associated mortality risk during drought compared with nondrought periods and found that increasing drought severity revealed a trend toward elevated mortality risk, although results did not achieve statistical significance as severe drought and heatwave conditions were relatively infrequent. Heatwave-associated mortality risks were also greater during drought and heatwave conditions in the late warm season, which contrasts with heatwave alone assessments.^17,49^ Limited research has assessed the public health impacts of compound extreme heat and drought events and few climate and health studies emphasize populations with existing respiratory disease.^17,30,31,50,51^

Individuals with existing respiratory diseases are at increased vulnerability to extreme weather events, including heatwaves and drought. For our cohort of older individuals diagnosed with COPD, plausible biological pathways relating extreme heat to COPD may include dehydration, impaired pulmonary perfusion, and airway inflammation.^52^ Individuals with COPD have shown increased breathlessness, inhaler use, and a worsening of overall symptoms during extreme heat exposure even after adjusting for air pollution.^53^ Additionally, people with COPD typically have multiple concomitant comorbidities including cardiovascular conditions, diabetes, and chronic kidney disease that deteriorate overall health, amplify sensitivity to extreme heat, and may increase the risk of heat-related injury, morbidity, or mortality.^54^ Older individuals are further prone to thermodysregulation and can suffer added cardiac strain due to the additional stress vasodilation places on the heart as the body attempts to cool itself when exposed to high temperatures.^55,56^

The mechanisms by which drought adversely affects respiratory health outcomes are less clear. Indirectly, drought worsens existing environmental hazards including air pollution (e.g., dust, wildfires, aeroallergens, and ozone) and extreme heat.^20–22^ The intensification of existing environmental exposures that are already detrimental to respiratory health most likely increases the risk of morbidity and mortality. Direct impacts of drought on respiratory outcomes are less understood and may act through mechanisms such as chronic stress. Drought is a long-lasting environmental exposure that can have ripple effects over a large geographic area affecting essential resources such as food and water availability, which can be a cause of stress particularly among economically disadvantaged and rural communities.^57^ Inflammation induced by chronic stress may worsen existing chronic health conditions or cause one to become more susceptible to infectious diseases via a weakened immune system. Indeed, drought conditions have been found to yield more favorable environments for certain foodborne and fungal diseases such as coccidioidomycosis to flourish.^57^ Overall, our results align with previous studies that found associations between drought and mortality.^1,16,32,33^

Compound drought and heat are a particularly critical climate hazard for North America, which has a historical record of pan-continental droughts and an increasing trend of elevated summer temperatures.^58,59^ Compound drought and heatwave events are hypothesized to emerge from complex land–atmosphere feedback loops. As soil and vegetation become desiccated, evapotranspiration declines and the air becomes drier, which reduces the likelihood of precipitation and favors the genesis of meteorological droughts.^23^ Meanwhile, as evapotranspiration declines, a larger fraction of incoming solar radiation warms the environment, which increases atmospheric heat that may generate a heatwave or amplify its intensity.^23^ Existing heat warnings may need to include drought conditions as an additional parameter to improve public health warning systems. Without the inclusion of drought data, warning systems may underestimate the true risk from heatwaves that occur during drought events. Other tools including heat vulnerability maps such as the recently released Heat and Health Index from the Centers for Disease Control and Prevention^60,61^ should account for drought conditions when characterizing community heat vulnerabilities.

The attribution of greater heatwave-associated mortality risk to late-season drought conditions departs from previous work on heatwaves alone. Prior research identified the initial heatwaves of the season having greater mortality risk under the hypothesis that the population has not physiologically acclimatized to warmer summer temperatures, placing them at greater susceptibility to early-season events.^17,49^ Our results were somewhat contrary to these findings. While early-season heatwaves engendered larger mortality risks compared with late-season heatwaves, there were no differences in mortality risk comparing drought and nondrought conditions. However, there was a relatively large difference in late-season heatwave-associated mortality comparing drought to nondrought conditions. Our data show similar increases in mean heatwave temperature comparing drought and nondrought periods for both early and late-season heatwaves. Yet, late-season heatwaves lasted longer than early-season heatwaves, particularly during drought. Therefore, it may be that the extended duration of late-season heatwaves during drought that is contributing to an elevated risk of mortality.

An additional consequence of increased climate-induced health events is the potential overburdening of existing healthcare infrastructure. It was estimated that extreme weather and climate-associated diseases in 2012, a year with widespread drought, intense heat, and ten hurricanes, cost the United States $10 billion in healthcare-related costs.^62^ Yet, the presence of compounding and cascading climate hazards has the potential to induce greater economic costs and loss of life than any single climate event alone. In North America, the summer of 2023 had several compounding climate disasters including the devastating wildfire in Maui, Hawaii that killed at least 97 people.^63^ This event occurred after a rapid 3-week flash drought, a phenomenon of drought and heat intensification that amplifies vegetation dryness and heightens conditions for severe wildfire events.^64^ The public health impacts of compounding events and their long-term effects have yet to be quantified but are essential to elucidate so health services can better prepare and adapt to this increasing threat.

Several limitations should be considered when interpreting our results. First, the generalizability of our study was limited, in part by, the demographic composition of our COPD cohort that was predominantly male, older, White race, and all veterans. However, we believe our findings on mortality risk from an acute environmental exposure should be generally representative of older nonveteran males. Our large cohort is predominantly older with most individuals decades removed from military service and residing across a national geography. A prior veteran status should have minimal impact on their mortality risk to acute environmental exposures of heatwaves and drought conditions. Second, our diagnosis of COPD was limited to ICD codes in electronic health records and while spirometry is considered the gold standard in COPD diagnosis, these data are not uniformly collected in the VHA system. Prior studies in the VA population report that ICD codes perform well with high specificity and moderate sensitivity.^65–67^ Third, despite having a relatively large study cohort, our study period was short, which contributed to the rarity of compound drought and heatwave events. With fewer patients simultaneously exposed to heatwaves and drought conditions, the precision of our estimates was affected, although a strong pattern of increasing risk with more severe drought and heatwaves was apparent. While our data prohibited evaluations by subgroups including age, gender, and race, these attributes should be explored in future work that may reveal additional health disparities of importance.

## Conclusion

Compound weather extremes of heatwaves and drought situated patients with existing respiratory disease at greater risk of death than patients experiencing heatwaves alone. These associations were more apparent in late-season heatwaves occurring under drought conditions. Developing an understanding of the public health impacts of complex climate events is needed for optimal climate change mitigation and adaptation strategies, particularly for vulnerable populations with existing respiratory diseases.

## Conflicts of interest statement

The authors declare that they have no conflicts of interest with regard to the content of this report.

## Acknowledgments

This material is the result of work supported with resources and the use of facilities at the Minneapolis VA Health Care System. We thank David Nelson, PhD, for providing statistical expertise.

## Supplementary Material
