# Supplementary material for: Compound drought and heatwave extreme weather events: Mortality risk in individuals with chronic respiratory disease
Source: Environ Epidemiol. 2025 May 1;9(3):e389. doi: 10.1097/EE9.0000000000000389 (PMC12045549; doi:10.1097/EE9.0000000000000389)
Supplement: Supplementary file 1 [file ee9-9-e389-s001.pdf]

Supplementary Materials to

Compound Drought and Heatwave Extreme Weather Events: Mortality Risk in Individuals with Chronic Respiratory Disease

Austin Rau[1], Arianne K Baldomero[2,3], Jesse E Bell[4,5,6], Jared Rennie[7], Chris H Wendt[2,3], Gillian AM Tarr[1], Bruce H Alexander[1], Jesse D Berman[1]

[1] University of Minnesota School of Public Health, Division of Environmental Health Sciences, Minneapolis, MN 55455, USA
[2] Minneapolis VA Health Care System, Pulmonary, Allergy, Critical Care, and Sleep Medicine Section, Minneapolis, MN 55417, USA
[3] University of Minnesota School of Medicine, Division of Pulmonary, Allergy, Critical Care, and Sleep Medicine, Minneapolis, MN 55455, USA
[4]University of Nebraska, Daugherty Water for Food Global Institute, Lincoln, NE 68501, USA
[5]University of Nebraska Medical Center, Department of Environmental, Agricultural and Occupational Health, Omaha, NE 68198, USA
[6]University of Nebraska-Lincoln, School of Natural Resources, Lincoln, NE 68583, USA
[7]National Oceanic and Atmospheric Administration, National Centers for Environmental Information, Asheville, NC 28801, USA

Corresponding Author:
Austin Rau
University of Minnesota School of Public Health
rauxx087@umn.edu

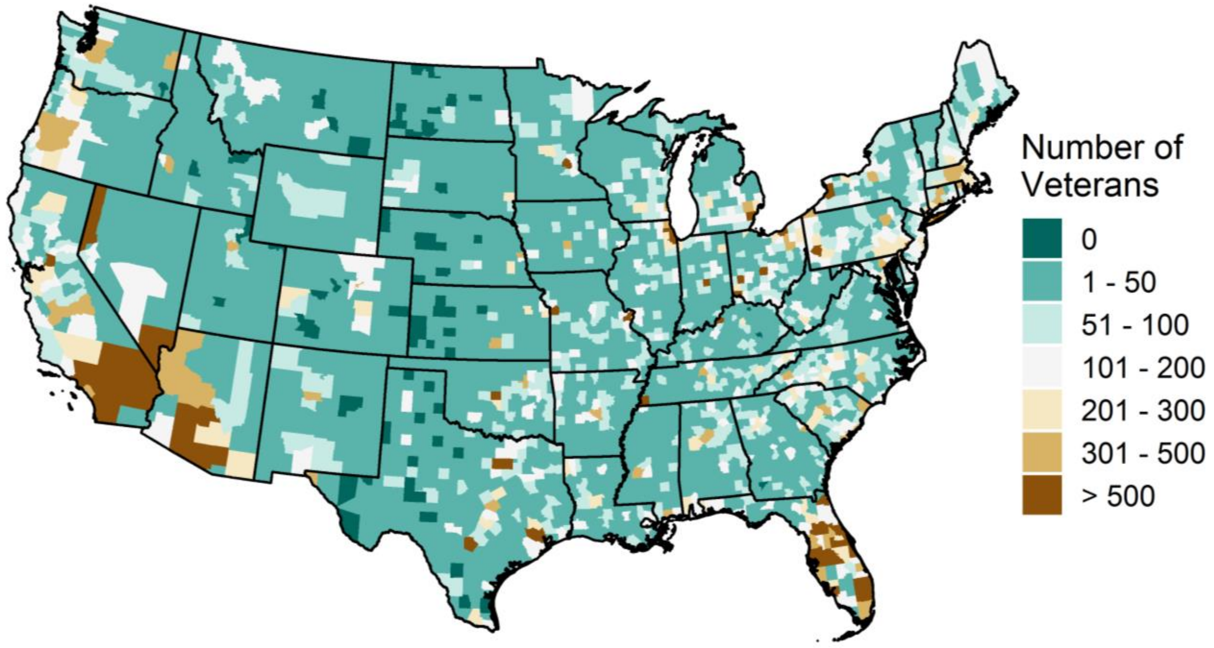

eFigure 1: County level distribution of deceased Veteran patients with COPD (2016 to 2021)

eTable 1: Demographic summary of deceased Veteran patients with COPD (2016 to 2021)

**Baseline Characteristic**	
Age (years), mean (SD)	76.9 (10.1)
Gender, frequency (%)	
Male	179,850 (97.9%)
Female	3,872 (2.1%)
Transgender	3 (<1%)
Race, frequency (%)	
White	145,575 (79.2%)
Black	20,987 (11.4%)
American Indian / Alaska Native	1,609 (<1%)
Asian American / Pacific Islander	1720 (<1%)
Missing	13,834 (7.5%)

eTable 2: Frequency of deceased COPD patients stratified by heatwave status, drought status, and timing of heatwaves

**Drought index**	**Timing**	**Heatwave status**	**Drought classification**	
			No Drought	Drought
USDM	Early	Non-heatwave, N (%)	78,293 (97.1%)	10,378 (94.6%)
		Heatwave, N (%)	2,366 (2.9%)	593 (5.4%)
		Total	80,659	10,971
	Late	Non-heatwave, N (%)	67,043 (84.9%)	10,390 (79.4%)
		Heatwave, N (%)	11,970 (15.1%)	2,692 (20.6%)
		Total	79,013	13,082
SPEI	Early	Non-heatwave, N (%)	67,987 (97.3%)	20,684 (95.1%)
		Heatwave, N (%)	1,899 (2.7%)	1,060 (4.9%)
		Total	69,886	21,744
	Late	Non-heatwave, N (%)	61,140 (85.7%)	16,293 (78.4%)
		Heatwave, N (%)	10,173 (14.3%)	4,489 (21.6%)
		Total	71,313	20,782

eTable 3: Mean heatwave temperature and duration on days of death for COPD patients stratified by binary drought categories and timing of heatwaves

**Drought index**	**Timing**	**Heatwave status**	**Drought classification**	
			No Drought	Drought
USDM	Early	Non-heatwave mean temperature °C (SD)	17.95 (6.94)	19.79 (6.39)
		Heatwave mean temperature °C (SD)	27.60 (2.68)	29.03 (4.04)
		Heatwave mean duration days (SD)	4.00 (2.32)	4.86 (3.22)
	Late	Non-heatwave mean temperature °C (SD)	23.25 (4.36)	22.52 (5.34)
		Heatwave mean temperature °C (SD)	27.85 (2.71)	28.20 (4.30)
		Heatwave mean duration days (SD)	5.02 (3.25)	6.75 (5.90)
SPEI	Early	Non-heatwave mean temperature °C (SD)	17.87 (6.92)	19.13 (6.76)
		Heatwave mean temperature °C (SD)	27.44 (2.46)	28.70 (3.77)
		Heatwave mean duration days (SD)	3.85 (2.20)	4.76 (2.99)
	Late	Non-heatwave mean temperature °C (SD)	23.04 (4.34)	23.57 (5.06)
		Heatwave mean temperature °C (SD)	27.54 (2.59)	28.75 (3.82)
		Heatwave mean duration days (SD)	4.84 (3.01)	6.46 (5.31)

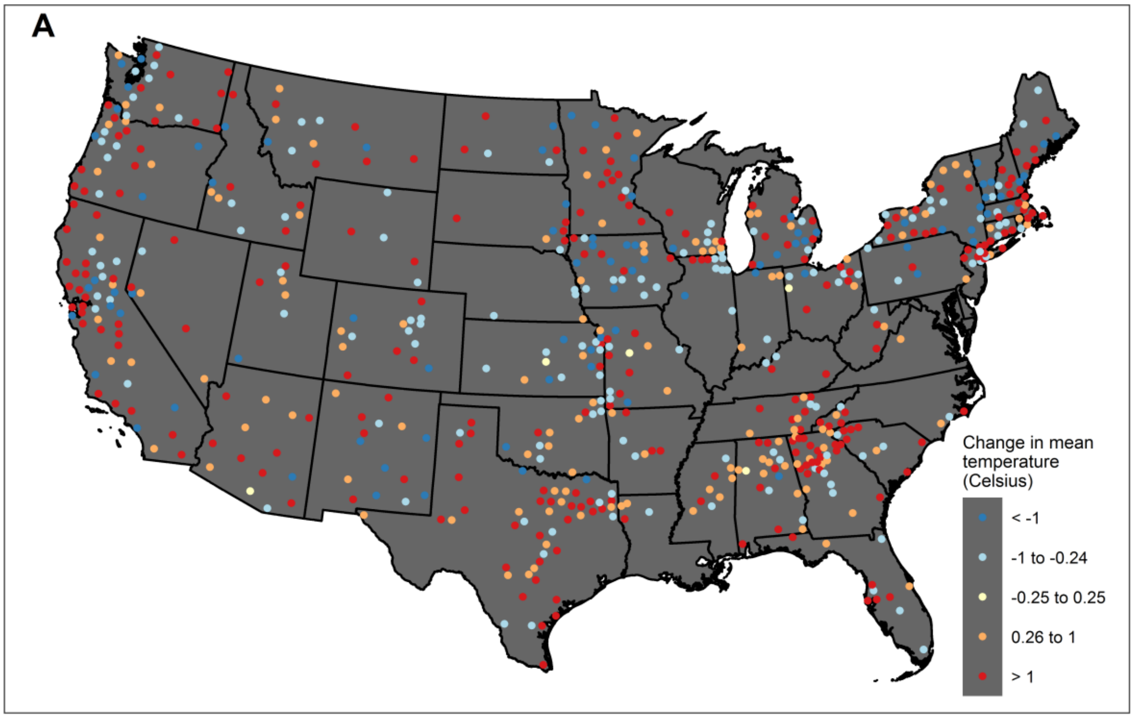

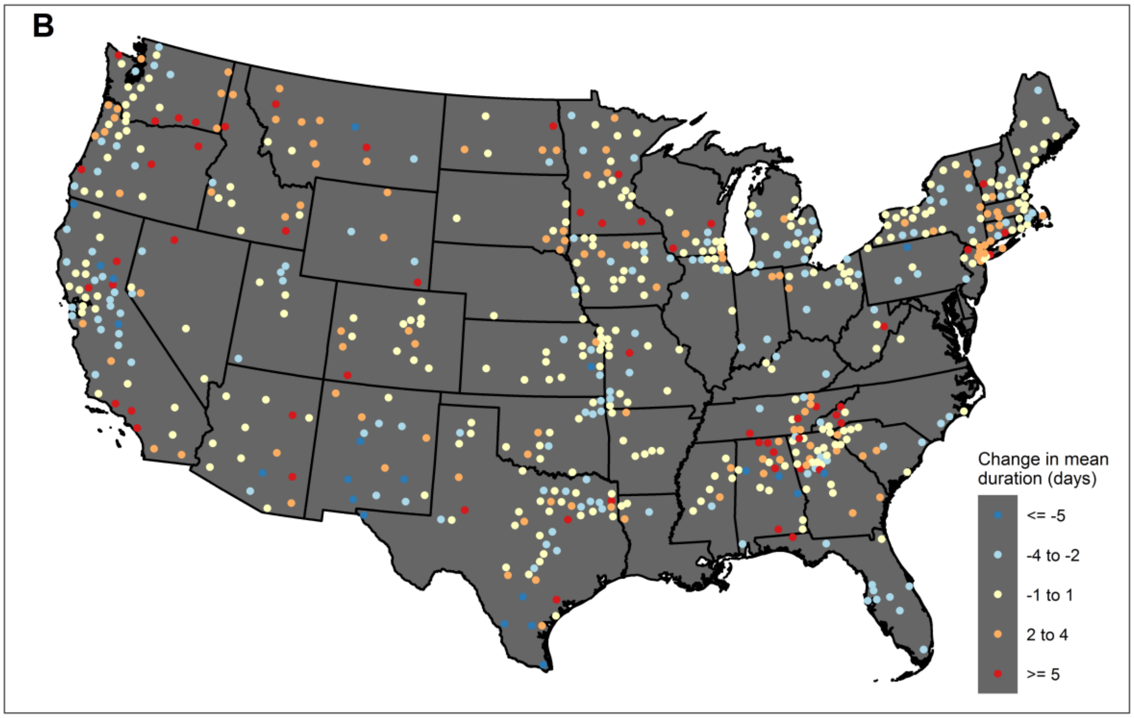

eFigure 2: Change in mean temperature (A) and mean duration (B) of heatwaves comparing drought to non-drought conditions on days of death for COPD patients. Dots represent county centroids (N = 622 counties that had patients with heatwave exposure during drought and non-drought conditions)

eTable 4: Adjusted incidence rate ratios describing the association between heatwaves and all-cause mortality among patients with COPD stratified by binary and categorical USDM drought definitions

Strata	Lag	IRR (95% CI)	P value
No drought (N = 159,672)	0	1.02 (1.00, 1.04)	Ref
	1	1.01 (1.00, 1.02)	Ref
	0 to 1	1.03 (1.00, 1.05)	Ref
Any drought (N = 24,053)	0	1.05 (1.02, 1.09)	0.10
	1	1.03 (1.01, 1.05)	0.11
	0 to 1	1.08 (1.03, 1.14)	0.09
Moderate drought (N = 18,858)	0	1.05 (1.01, 1.10)	0.13
	1	1.02 (1.00, 1.05)	0.23
	0 to 1	1.08 (1.02, 1.15)	0.15
Severe drought (N = 5,195)	0	1.05 (0.98, 1.13)	0.40
	1	1.03 (1.00, 1.07)	0.20
	0 to 1	1.09 (0.98, 1.21)	0.31

eTable 5: Adjusted incidence rate ratios describing the association between heatwaves and all-cause mortality among patients with COPD stratified by binary USDM drought definitions and timing of heatwaves

Strata (N)	Lag	IRR (95% CI)	P value[a]
Early season, no drought (N = 80,659)	0	1.06 (1.02, 1.10)	Ref
	1	1.03 (1.01, 1.05)	Ref
	0 to 1	1.10 (1.04, 1.16)	Ref
Early season, drought (N = 10,971)	0	1.05 (0.97, 1.14)	0.82
	1	1.03 (0.99, 1.07)	0.97
	0 to 1	1.09 (0.97, 1.22)	0.89
Late season, no drought (N = 79,013)	0	1.01 (0.99, 1.03)	Ref
	1	1.00 (0.99, 1.01)	Ref
	0 to 1	1.01 (0.99, 1.04)	Ref
Late season, drought (N = 13,082)	0	1.05 (1.01, 1.10)	0.06
	1	1.03 (1.00, 1.05)	0.09
	0 to 1	1.08 (1.02, 1.15)	0.06

[a]Early season no drought is the referent compared to early season drought and late season no drought is the referent compared to late season drought estimates.

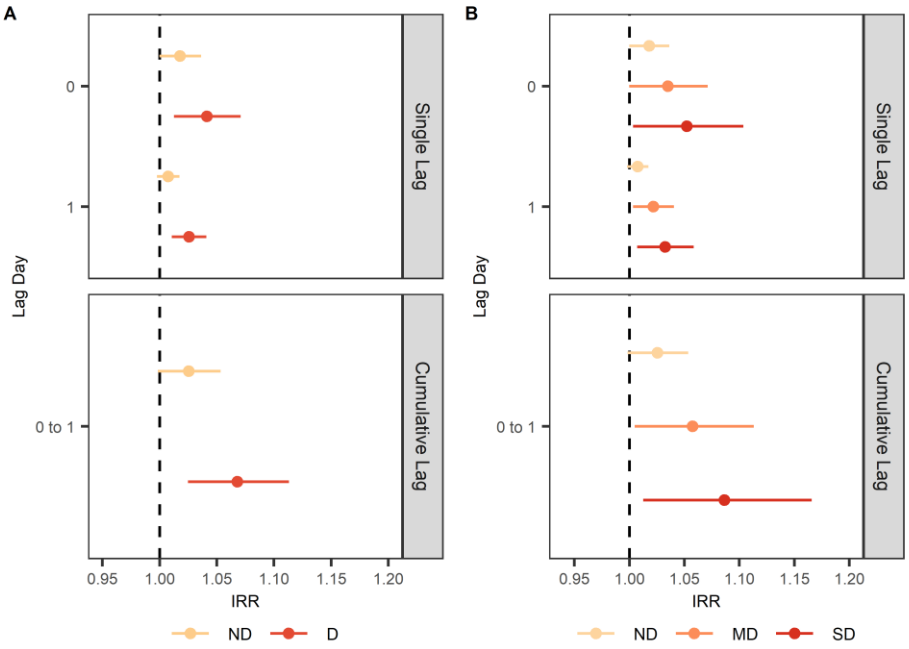

eFigure 3: Adjusted incidence rate ratios (IRR) describing the association between heatwaves and all-cause mortality among patients with COPD stratified by binary (A) and categorical (B) SPEI drought definitions. ND = No drought, D = Drought, MD = Moderate drought, SD = Severe drought.

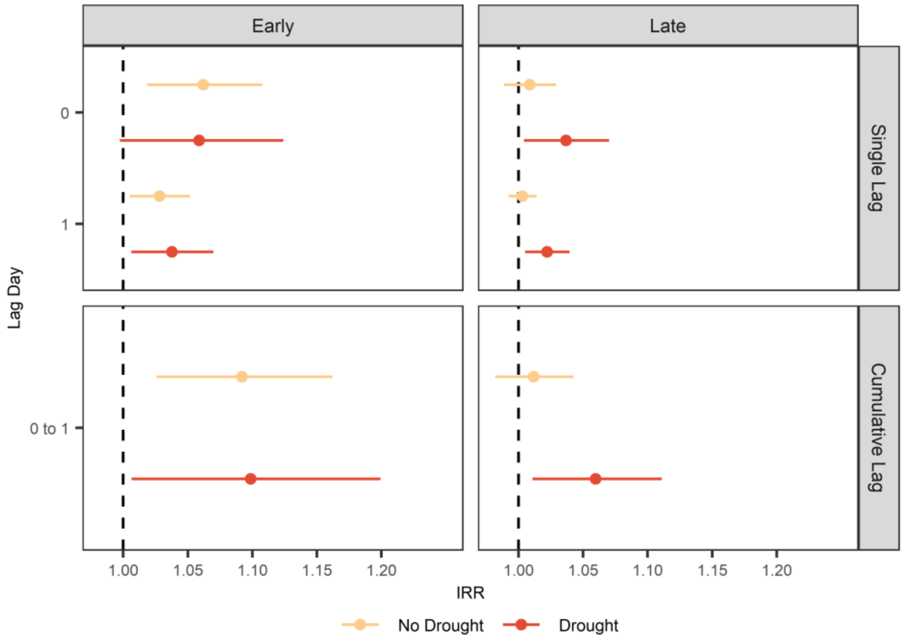

eFigure 4: Adjusted incidence rate ratios (IRR) describing the association between heatwaves and all-cause mortality among patients with COPD stratified by binary SPEI drought definitions and timing of heatwaves (early = April to June and late = July to September)

eTable 6: Adjusted incidence rate ratios describing the association between heatwaves and all-cause mortality among patients with COPD stratified by binary and categorical SPEI drought definitions

Strata (N)	Lag	IRR (95% CI)	P value
No drought (N = 141,199)	0	1.02 (1.00, 1.04)	Ref
	1	1.01 (1.00, 1.02)	Ref
	0 to 1	1.03 (1.00, 1.05)	Ref
Any drought (N = 42,526)	0	1.04 (1.01, 1.07)	0.18
	1	1.03 (1.01, 1.04)	0.05
	0 to 1	1.07 (1.02, 1.11)	0.11
Moderate drought (N = 29,421)	0	1.04 (1.00, 1.07)	0.40
	1	1.02 (1.00, 1.04)	0.18
	0 to 1	1.06 (1.00, 1.11)	0.30
Severe drought (N = 13,105)	0	1.05 (1.00, 1.10)	0.20
	1	1.03 (1.01, 1.06)	0.07
	0 to 1	1.09 (1.01, 1.17)	0.13

eTable 7: Attributable risk (%) for the cumulative (lag 0 to 1) heatwave associations stratified by SPEI drought (binary and categorical) and by timing of heatwaves

Strata	AR% (95% CI)
No drought	2.91 (0.00, 4.76)
Drought	6.54 (1.96, 9.91)
Moderate drought	5.66 (0.00, 9.91)
Severe drought	8.26 (0.99, 14.53)
Heatwave timing	
Early (No Drought)	8.26 (2.91, 13.79)
Early (Drought)	9.09 (0.99, 16.67)
Late (No Drought)	0.99 (-2.04, 3.85)
Late (Drought)	5.66 (0.99, 9.91)

eTable 8: Adjusted incidence rate ratios describing the association between heatwaves and all-cause mortality among patients with COPD stratified by binary SPEI drought and timing of heatwaves

**Strata (N)**	**Lag**	**IRR (95% CI)**	**P value**[a]
Early season, no drought (N = 69,886)	0	1.06 (1.02, 1.11)	Ref
	1	1.03 (1.01, 1.05)	Ref
	0 to 1	1.09 (1.03, 1.16)	Ref
Early season, drought (N = 21,744)	0	1.06 (1.00, 1.12)	0.94
	1	1.04 (1.01, 1.07)	0.63
	0 to 1	1.10 (1.01, 1.20)	0.91
Late season, no drought (N = 71,313)	0	1.01 (0.99, 1.03)	Ref
	1	1.00 (0.99, 1.01)	Ref
	0 to 1	1.01 (0.98, 1.04)	Ref
Late season, drought (N = 20,782)	0	1.04 (1.00, 1.07)	0.06
	1	1.02 (1.01, 1.04)	0.09
	0 to 1	1.06 (1.01, 1.11)	0.06

[a]Early season no drought is the referent compared to early season drought and late season no drought is the referent compared to late season drought estimates.

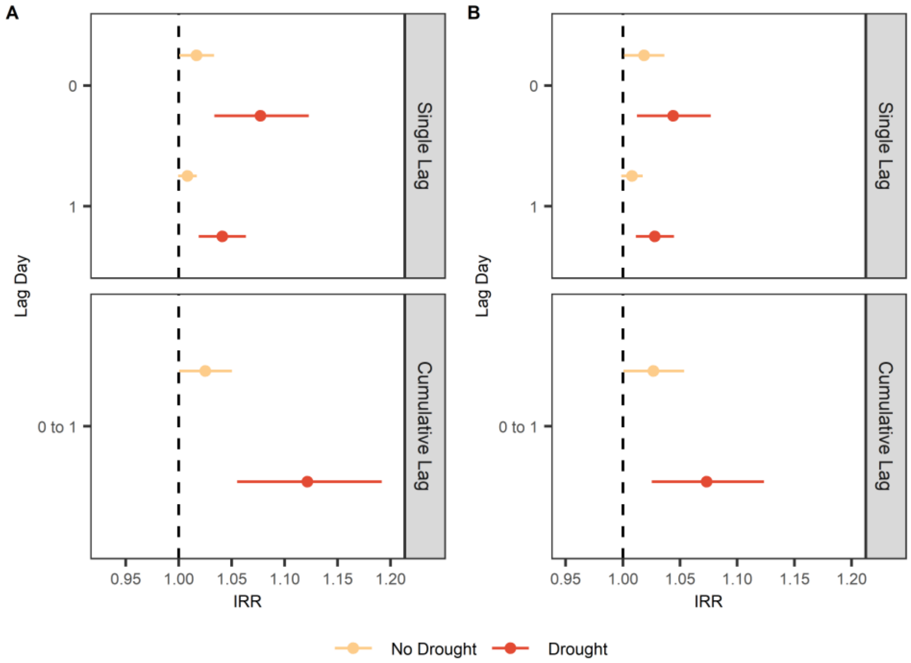

eFigure 5: Adjusted incidence rate ratios (IRR) describing the association between heatwaves and all-cause mortality among patients with COPD stratified by binary A) USDM and B) SPEI. Drought events were ≥5 consecutive months of at least D1 or higher

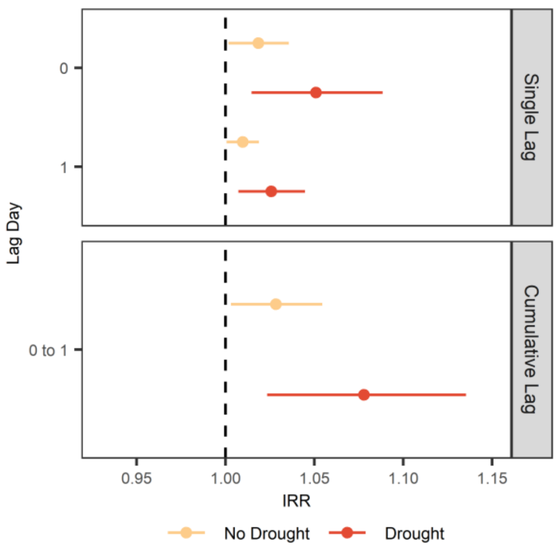

eFigure 6: Adjusted incidence rate ratios (IRR) describing the association between heatwaves and all-cause mortality among patients with COPD stratified by binary USDM drought definition where drought exposure was defined using weekly USDM data
